# Sustained effect of prasinezumab on Parkinson’s disease motor progression in the open-label extension of the PASADENA trial

**DOI:** 10.1038/s41591-024-03270-6

**Published:** 2024-10-08

**Authors:** Gennaro Pagano, Annabelle Monnet, Adriana Reyes, Benjamin Ribba, Hanno Svoboda, Thomas Kustermann, Tanya Simuni, Ronald B. Postuma, Nicola Pavese, Fabrizio Stocchi, Kathrin Brockmann, Krzysztof Smigorski, Valentina Gerbaldo, Paulo Fontoura, Rachelle Doody, Geoffrey A. Kerchner, Patrik Brundin, Kenneth Marek, Azad Bonni, Tania Nikolcheva, PASADENA Investigators, Tanya Simuni, Kathrin Brockmann, Prasinezumab Study Group, Gennaro Pagano, Annabelle Monnet, Adriana Reyes, Benjamin Ribba, Hanno Svoboda, Thomas Kustermann, Krzysztof Smigorski, Paulo Fontoura, Rachelle Doody, Geoffrey A. Kerchner, Patrik Brundin, Azad Bonni, Tania Nikolcheva

**Affiliations:** 1 Roche Pharma Research and Early Development (pRED), Neuroscience and Rare Diseases Discovery and Translational Area, Roche Innovation Center Basel, Basel, Switzerland; 2 University of Exeter Medical School, London, UK; 3 F. Hoffmann-La Roche Ltd, Basel, Switzerland; 4 Roche Diagnostics GmbH, Penzberg, Germany; 5 Department of Neurology, Northwestern University Feinberg School of Medicine, Chicago, IL USA; 6 Department of Neurology, McGill University and Montreal Neurological Institute, Montreal, Quebec Canada; 7 Clinical Ageing Research Unit, Newcastle University, Newcastle upon Tyne, UK; 8 University San Raffaele Roma and the Institute for Research and Medical Care, IRCCS San Raffaele Pisana, Rome, Italy; 9 Hertie Institute for Clinical Brain Research and German Center for Neurodegenerative Diseases (DZNE), University of Tuebingen, Tuebingen, Germany; 10 Excelya Germany GmbH, Freiburg, Germany; 11 Genentech USA Inc., San Francisco, CA USA; 12 Institute for Neurodegenerative Disorders, New Haven, CT USA

**Keywords:** Motor control, Parkinson's disease

## Abstract

The Phase II trial of Anti-alpha-Synuclein Antibody in Early Parkinson’s Disease (PASADENA) is an ongoing double-blind, placebo-controlled trial evaluating the safety and efficacy of prasinezumab in early-stage Parkinson’s disease (PD). During the double-blind period, prasinezumab-treated individuals showed less progression of motor signs (Movement Disorders Society-sponsored revision of the Unified Parkinson’s Disease Rating Scale (MDS–UPDRS) Part III) than placebo-treated individuals. We evaluated whether the effect of prasinezumab on motor progression, assessed as a change in MDS–UPDRS Part III score in the OFF and ON states, and MDS–UPDRS Part II score, was sustained for 4 years from the start of the trial. We compared participants enrolled in the PASADENA open-label extension study with those enrolled in an external comparator arm derived from the Parkinson’s Progression Markers Initiative observational study. The PASADENA delayed-start (*n* = 94) and early-start (*n* = 177) groups showed a slower decline (a smaller increase in score) in MDS–UPDRS Part III scores in the OFF state (delayed start, −51%; early start, −65%), ON state (delayed start, −94%; early start, −118%) and MDS–UPDRS Part II (delayed start, −48%; early start, −40%) than did the Parkinson’s Progression Markers Initiative external comparator (*n* = 303). This exploratory analysis, which requires confirmation in future studies, suggested that the effect of prasinezumab in slowing motor progression in PD may be sustained long term. PASADENA ClinicalTrials.gov no. NCT03100149.

## Abstract (web summary)

Individuals with Parkinson’s disease treated with prasinezumab demonstrated slower motor progression over 4 years compared with an external comparator cohort derived from the Parkinson’s Progression Markers Initiative observational study.

## Main

Prasinezumab is a humanized monoclonal antibody designed to bind aggregated α-synuclein and inhibit the intercellular spread of pathogenic α-synuclein, thus potentially protecting neurons and slowing Parkinson’s disease (PD) progression^1–3^.

The Phase II trial of Anti-alpha-Synuclein Antibody in Early Pakinson’s Disease (PASADENA; NCT03100149) is an ongoing multicenter, randomized, double-blind, placebo-controlled trial evaluating the safety and efficacy of intravenous prasinezumab administered every 4 weeks in early-stage PD^4^. The study is divided into three parts: a 12-month double-blind period during which participants were treated with 1,500 mg of prasinezumab and 4,500 mg of prasinezumab or placebo (Part 1); a 12-month period during which participants treated with placebo were rerandomized to either 1,500 or 4,500 mg of prasinezumab while prasinezumab-treated participants continued on their dose of prasinezumab (Part 2); and a long-term (5 years) open-label extension (OLE) in which all participants received 1,500 mg of prasinezumab (Part 3).

In the double-blind study period (Part 1), the primary endpoint was not met (Movement Disorders Society-sponsored revision of the Unified Parkinson’s Disease Rating Scale (MDS–UPDRS) sum of Parts I, II and III)^4^. However, prasinezumab-treated individuals (both low- and high-dose) showed less motor progression in MDS–UPDRS Part III than placebo-treated individuals^4^. The OLE (Part 3) was implemented as an amendment to the PASADENA study following the completion of Part 1, to evaluate the long-term safety and efficacy of prasinezumab.

**Fig. 1:**
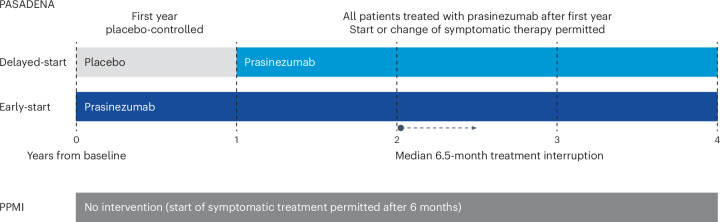
Timeframe for analysis for PASADENA and PPMI. In PASADENA, the delayed-start group received placebo during the first year of the trial (light gray bar), after which they were switched to prasinezumab (light blue bar). The early-start group (dark blue bar) received prasinezumab for the whole duration of the trial. The PPMI study (dark gray bar) received no intervention.

Here, we performed an exploratory analysis comparing participants treated with prasinezumab in the PASADENA OLE with subjects enrolled in the Parkinson’s Progression Markers Initiative (PPMI) observational study^5^, in which an external comparator arm was created by means of two independent approaches: propensity score adjustment and disease modeling. The PPMI was used to put into context the rates of disease progression seen in the OLE of PASADENA, in the absence of a placebo group beyond PASADENA Part 1 (Fig. 1).

The main objective of the present study was to evaluate the change in severity of motor progression from baseline to year 4 in the PASADENA OLE cohort versus the external comparator population.

## Results

Baseline characteristics of the PASADENA and PPMI cohorts were well balanced after weighting with propensity scores (Table 1).

**Table 1:** Baseline characteristics before and after weighting with propensity scores

Characteristic	Before weighting			After weighting		
	PASADENA, *n* = 271	EC PPMI, *n* = 303	SMD	PASADENA, *n* = 271	EC PPMI, *n* = 270	SMD
Age (years), mean (s.d.)	59.98 (9.00)	62.11 (8.53)	0.243	59.98 (9.00)	61.20 (9.28)	0.133
Male sex, *n* (%)	188 (69.4)	202 (66.7)	0.058	188.0 (69.4)	189.3 (70.1)	0.017
MDS–UPDRS Part III, mean (s.d.)	21.15 (8.96)	21.17 (8.85)	0.003	21.15 (8.96)	21.13 (9.71)	0.001
H&Y 2, *n* (%)	201 (74.2)	183 (60.4)	0.297	201.0 (74.2)	205.7 (76.2)	0.047
PD diagnosis (months), mean (s.d.)	9.89 (6.34)	4.87 (5.36)	0.855	9.89 (6.34)	9.20 (5.61)	0.115
Years of education ≥12, *n* (%)	244 (90.0)	279 (92.1)	0.072	244.0 (90.0)	236.2 (87.5)	0.080
Montreal Cognitive Assessment, mean (s.d.)	28.17 (1.79)	27.23 (2.26)	0.462	28.17 (1.79)	28.02 (1.89)	0.082
DaT–SPECT putamen bilateral, mean (s.d.)	0.92 (0.26)	0.81 (0.28)	0.436	0.92 (0.26)	0.92 (0.31)	0.018

Note: SMD ≤ 0.2 indicates balance between groups. EC, external control.

**Fig. 2:**
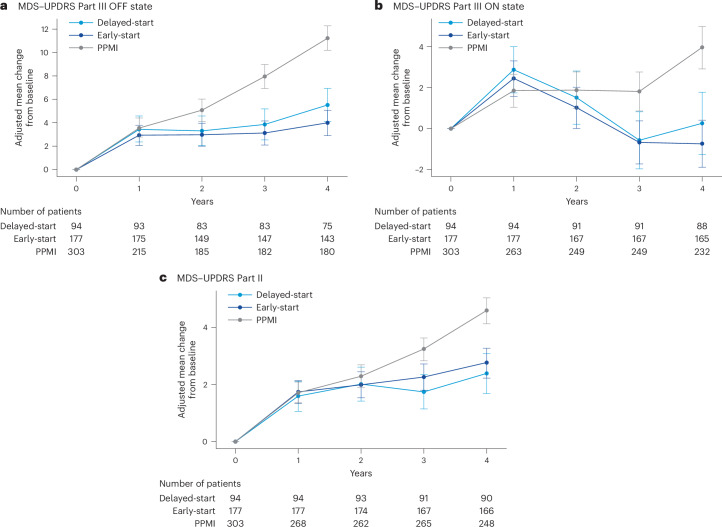
Adjusted mean change from baseline in MDS–UPDRS Part III in ON and OFF states and MDS–UPDRS Part II. **a**, MDS–UPDRS Part III in OFF state. **b**, MDS–UPDRS Part III in ON state. **c**, MDS–UPDRS Part II. Error bars represent 80% CI.

In comparison with those in the PPMI cohort, at year 4 the PASADENA delayed- and early-start groups both had lower MDS–UPDRS Part III progression in the OFF state, with a −51% relative difference (mean (80% confidence interval (CI)), −5.73 (−7.33 to −4.14) points) for the delayed-start group and a −65% relative difference (mean (80% CI), −7.26 (−8.59 to −5.93) points) for the early-start group (Fig. 2a); lower MDS–UPDRS Part III progression in the ON state, with a −94% relative difference (mean (80% CI), −3.71 (−5.41 to −2.01) points) for the delayed-start group and a −118% relative difference (mean (80% CI), −4.69 (−6.09 to −3.3) points) for the early-start group (Fig. 2b); and lower MDS–UPDRS Part II progression, with a −48% relative difference (mean (80% CI), −2.20 (−2.96 to −1.45) points) for the delayed-start group and −40% relative difference (mean (80% CI), −1.82 (−2.44 to −1.2) points) for the early-start group (Fig. 2c).

**Fig. 3:**
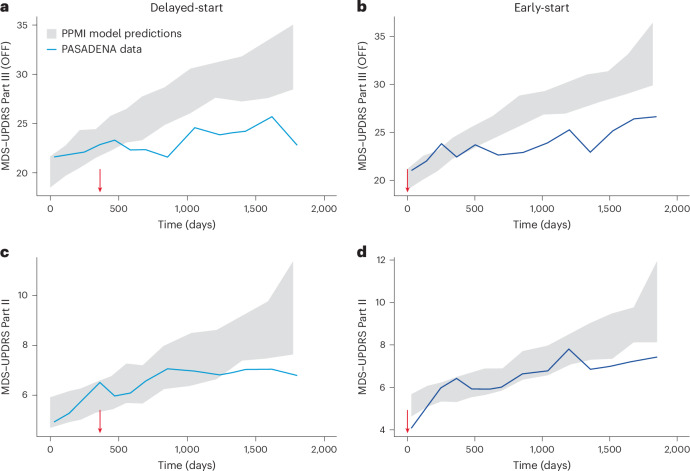
PPMI-based median prediction CIs overlayed with PASADENA median data. **a**, MDS–UPDRS Part III OFF delayed-start group (empirical data represented in light blue). **b**, MDS–UPDRS Part III OFF early-start group (dark blue). **c**, MDS–UPDRS Part II delayed-start group (empirical data represented in light blue). **d**, MDS–UPDRS Part II early-start group (dark blue). Vertical red arrows indicate the start of prasinezumab treatment for both groups. Gray areas represent PPMI-based median prediction CIs.

Disease progression modeling showed findings similar to those obtained with the propensity score method. The MDS–UPDRS Part III (OFF) and Part II scores in the PASADENA delayed- and early-start groups were below the 90% CI for the respective values calculated using the data from the PPMI cohort (Fig. 3). Separation from the PPMI-based prediction occurred from year 2 onwards for MDS–UPDRS Part III and during year 4 for MDS–UPDRS Part II. In the analysis of MDS–UPDRS Part III score in the OFF state, 55% of PASADENA scores were below the median PPMI predictions in year 2, and 64 and 66% were below the median PPMI predictions in years 3 and 4, respectively. For MDS–UPDRS Part II, the percentage of PASADENA scores below the median for PPMI predictions was close to 50% during years 2 and 3 (51 and 53%, respectively) but reached 56% below the median during year 4, which is comparable to the deviation of Part III in the OFF state during year 2. The percentage of MDS–UPDRS Part II scores below the median for PPMI predictions continued to increase thereafter, reaching 59% after year 4.

**Extended Data Fig. 1:**
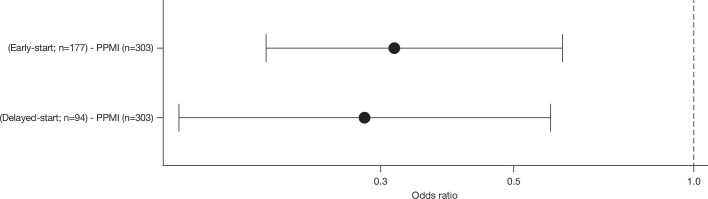
Adjusted logistic model Hoehn & Yahr ≥3 at year 4. Error bars represent 80% CI. Number of patients at baseline: delayed-start *n* = 94; early-start *n* = 177; PPMI; *n* = 303. CI, confidence interval; PPMI, Parkinson’s Progression Markers Initiative.

There was no difference in MDS–UPDRS Part IV progression between the PASADENA and PPMI cohorts (Extended Data Table 1). The odds ratio of having reached stage 3 or above in Hoehn and Yahr (H&Y) at year 4 was 0.26 (80% CI 0.04–0.42) in the PASADENA delayed-start group and 0.3 (80% CI 0.13–0.47) in the PASADENA early-start group, in comparison with the PPMI cohort (Extended Data Fig. 1).

**Extended Data Fig. 2:**
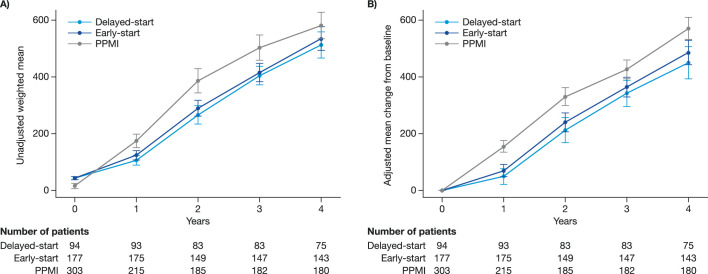
LEDD change over time. (**a**) LEDD plot of mean and s.d. over time; (**b**) LEDD MMRM change from baseline. Error bars represent 80% CI. CI, confidence interval; LEDD, levodopa equivalent daily dose; MMRM, Mixed Model for Repeated Measures; PPMI, Parkinson’s Progression Markers Initiative; s.d., standard deviation.

The PASADENA delayed- and early-start groups and the PPMI cohort showed similar use of symptomatic therapies over the 4 years (Extended Data Table 2). The PASADENA delayed- and early-start groups showed numerically lower levodopa equivalent daily dose (LEDD) intake at year 4 than did the PPMI cohort: mean −120.83 mg (80% CI −187.68 to −53.99) for the delayed-start group and mean −85.08 mg (80% CI −140.01 to −30.16) for the early-start group (Extended Data Fig. 2).

**Extended Data Fig. 3:**
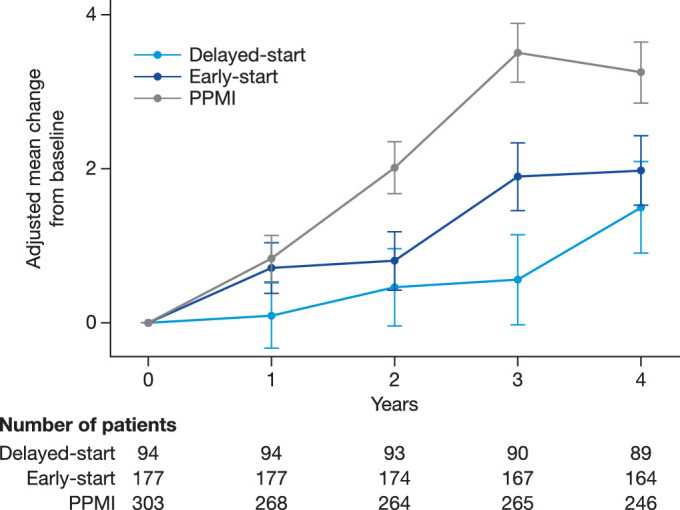
Change from baseline for MDS-UPDRS Part I of PASADENA (early- and delayed-start) against PPMI. Error bars represent 80% CI. CI, confidence interval; MDS-UPDRS, Movement Disorder Society-sponsored revision of the Unified Parkinson’s Disease Rating Scale; PASADENA, Phase II trial of Anti alpha-Synuclein AntiboDy in Early ParkiNson's diseAse; PPMI, Parkinson’s Progression Markers Initiative.

For MDS–UPDRS Part I total score, the PASADENA delayed- and early-start groups showed a mean progression after 4 years of 6.28 points (80% CI 5.67–6.89) and 6.39 (80% CI 5.87–6.91), respectively, while the PPMI cohort showed a mean progression of 8.34 points (80% CI 7.91–8.77) (Extended Data Fig. 3). The PASADENA delayed- and early-start groups also had lower MDS–UPDRS Part I Sleep progression scores (items 7 and 8) than did the PPMI cohort, with a −47% relative difference (mean (80% CI), −0.24 (−0.39 to −0.09) points) for the delayed-start group and a −61% relative difference (mean (80% CI), −0.31 (−0.43 to −0.19) points) for the early-start group after 4 years. Notably, in comparison with those in the PPMI cohort, the PASADENA delayed- and early-start groups showed no difference in MDS–UPDRS Part I Fatigue (item 13) after 4 years: mean −0.11 points (80% CI −0.2 to −0.02) for the delayed-start group and mean −0.06 points (80% CI −0.13 to 0.01) for the early-start group.

In comparison with the PPMI cohort, the PASADENA delayed- and early-start groups showed no difference in dopamine transporter imaging with single-photon emission computed tomography (DaT–SPECT) putamen or caudate striatal binding ratio progression over 4 years: mean 0.02 points (80% CI 0–0.06) for the delayed-start group and mean 0.03 points (80% CI 0–0.05) for the early-start group for the putamen bilateral striatal binding ratio.

A sensitivity analysis of MDS–UPDRS Parts II and III was performed, comparing the PPMI cohort with the subset (around 60%) of PASADENA participants who were treatment naive at baseline (excluding participants taking monoamine oxidase type B (MAO-B) inhibitors at baseline). The results were consistent with those obtained when all PASADENA participants were included (Supplementary Results and Supplementary Fig. 1).

## Discussion

In this exploratory analysis of the PASADENA study, people with PD treated with prasinezumab showed slower motor progression, as measured by MDS–UPDRS Part III (total and subscores) in the OFF and ON medication states, and Part II over 4 years, compared with an external comparator cohort derived from the PPMI observational study and a mathematical model of PD progression. Only low numbers of participants reached H&Y stage 3 (mild to moderate bilateral involvement, some postural instability but physical independence), both among PASADENA participants and in the PPMI comparator cohort, but prasinezumab-treated individuals still showed a lower risk of developing balance issues at year 4.

During the past two decades, evidence from genetic, neuropathological and experimental models has suggested that α-synuclein aggregates play an important role in the pathogenesis of PD^6^. The observation that α-synuclein aggregation may spread intercellularly and contribute to neurodegeneration supported the development of immunotherapies targeting α-synuclein aggregates. Prasinezumab is a humanized monoclonal antibody directed against aggregated α-synuclein, and its impact on PD progression was studied in the PASADENA Phase II study^4^. Although PASADENA did not meet its primary endpoint (change from baseline in the sum of MDS–UPDRS Parts I, II and III scores) at week 52, prasinezumab reduced the decline in motor function as measured by changes from baseline in the MDS–UPDRS Part III score and the use of digital readouts, at week 52 (ref. ^4^). The PASADENA OLE provided the opportunity to study the long-term effect of prasinezumab. Notably, >85% of PASADENA participants elected to enroll in the OLE and continued with monthly infusions of prasinezumab for 4 years. A major limitation of the OLE is the lack of a placebo arm, and, for this reason, it is not possible to fully rule out a potential placebo effect as an explanation for the differences in progression between PASADENA participants and the PPMI cohort. We utilized the PPMI observational arm as a suitable comparator to enable quantification of treatment effects in the absence of a placebo arm beyond the first 52 weeks of the study. Using a well-established method of propensity score balancing of baseline characteristics, we matched the trial and comparator groups^7,8^. The use of weighting facilitates the utilization of more covariates (including continuous ones) and includes all participants in the analysis. To ensure comparability, we applied the full PASADENA inclusion/exclusion criteria when selecting the PPMI cohort, and we next applied weighting (as opposed to matching) due to its flexibility in handling covariates. The validity of this approach for this study was confirmed by similar baseline characteristics and progression over 52 weeks between the PASADENA Part 1 placebo cohort and the weighted PPMI cohort.

As expected, in the present study, the PPMI cohort exhibited a rate of progression consistent with that reported in previous publications^9^. In contrast, the PASADENA delayed- and early-start prasinezumab-treated groups showed less worsening of MDS–UPDRS Part III in OFF state total score and subscores, MDS–UPDRS Part III in the ON state and MDS–UPDRS Part II. We confirmed this finding using a disease-modeling approach in which we compared PASADENA OLE data with a hypothetical population created by using the characteristics of PASADENA and a model of progression^10^. Despite the lack of a placebo arm, it is unlikely that these results are related to a placebo effect. First, the curves reflecting disease progression (both motor and daily function) in the PASADENA OLE arms and PPMI observational cohort did not show an immediate separation, but instead exhibited a gradually increasing degree of separation that continued between years 3 and 4. This is consistent with the item-response models of drugs with a potential disease-modifying effect^11^. Second, the observed differences are large (reduction in progression ranging from −65 to −118%). To help interpret these group differences, within-patient change estimates can be used to demonstrate that the average patient on treatment has a meaningfully better outcome than the average patient in the comparator group^12^; however, these may overestimate minimal meaningful group differences^13^. Therefore, group differences might still be meaningful when below this threshold but there is greater certainty that they are meaningful when above it. A within-patient worsening threshold of 5 points has been identified for MDS–UPDRS Part III OFF^14,15^, and thus the observed differences in change scores are meaningful. Even in participants meeting objectively defined criteria of placebo response in terms of motor signs (MDS–UPDRS Part III), subjective perception of daily function is relatively rarely seen^16^. Notably, at the 4-year time point our data from the OLE show a difference of around 6‒7 points in MDS–UPDRS Part III OFF, with the difference growing by around 3 points between years 3 and 4. Third, between years 2 and 3 there was a treatment-free washout period of, on average, 7.4 months (median 6.5 months). Despite this extended treatment-free period, the subsequent data points did not show clear worsening (which would have been predicted had the differences been due to a placebo effect), further reducing the probability that the difference is primarily due to a placebo effect.

We did not observe a difference in the rate of change in the DaT–SPECT measurements. There could be a number of explanations for this. It is plausible that prasinezumab may preserve dopaminergic synaptic function, thus translating to the observed clinical benefit but not impacting transporter function as measured by DaT–SPECT. DaT–SPECT availability is also influenced by compensatory downregulation, which can mask the effective progressive loss of terminals. Notably, several studies suggest that DaT scan imaging cannot be used as a fully quantitative biomarker to test treatment effect in PD and does not reflect overall disease severity^17^. Furthermore, previous work has shown that DaT scan results do not correlate with the number of tyrosine hydroxylase-positive axons in the putamen at autopsy^18^. The DaT–SPECT findings could also be explained by suboptimal matching of the DaT–SPECT signal in the putamen. Matching of the PPMI and PASADENA groups was only partially achieved for the DaT–SPECT signal. Although the baseline difference was balanced (standardized mean difference (SMD) <0.2), there was a substantial difference in the 1-year decline between the PPMI and PASADENA delayed-start (placebo) groups. Thus, the PPMI and PASADENA cohorts were not fully matched in terms of DaT–SPECT progression. It is unclear what drove the 1-year differences between cohorts, but substantial variability in progression rates of DaT–SPECT signals among cohorts has been reported^19,20^. We did not observe a difference in MDS–UPDRS Part IV scores, but participants started symptomatic therapy at different points, contributing to variability. A longer follow-up on stable symptomatic therapy is needed to assess the potential effect of prasinezumab on motor complications. At the 4-year time point, people with PD treated with prasinezumab had progressed less regarding nonmotor symptoms—as measured by MDS–UPDRS Part I (total and sleep subscores)—than the external comparator cohort derived from participants selected from the PPMI cohort. However, at the 1-year time point, the PASADENA delayed-start group (placebo until the end of year 1) already had a lower total score on MDS–UPDRS Part I than did the selected PPMI cohort; therefore, the difference observed at 4 years should be interpreted with great caution. Among the nonmotor symptoms of PD, sleep was prespecified in this analysis because sleep problems are among the most specific nonmotor symptoms of PD and are associated with poor quality of life^21^.

Both the PASADENA and PPMI studies enrolled treatment-naive participants, and PASADENA also included participants on stable doses of MAO-B inhibitors at baseline. While participants in both studies were instructed not to start or change symptomatic therapy for the first 6 months (PPMI) or 12 months (PASADENA), all participants were expected to be on symptomatic treatment eventually. A change in medication represents one of the strongest confounders when assessing PD progression, due to its effect on motor outcome measures. Therefore, we compared the types and doses of symptomatic medications across the PASADENA and PPMI cohorts. Despite slight differences between the two studies during the first 12 months, by year 2 both cohorts were similar in terms of the type of PD medication used and LEDD, suggesting that these differences are unlikely to explain the differences in the trajectories of progression of MDS–UPDRS Parts III and II scores. At years 3 and 4, when >90% of PASADENA and PPMI participants were receiving levodopa and/or dopamine receptor agonists, prasinezumab-treated participants exhibited lower MDS–UPDRS Part III motor scores in the ON state than did both the PPMI cohort and their own scores at baseline. In addition, the average LEDDs in the PASADENA arms were numerically lower than in the PPMI cohort. These findings suggested that prasinezumab could synergize with dopaminergic medications. It has previously been proposed that removal of extracellular α-synuclein aggregates might improve synaptic transmission in the nigrostriatal system, in addition to reducing the spread of protein aggregates between neurons^6^. There was a gap between the completion of Part 2 and initiation of the OLE phase of the study, which averaged 7.4 months and was largely driven by COVID-19-induced delays and other study operational aspects. Nevertheless, there was a clear separation between the PASADENA groups and the PPMI cohort on MDS–UPDRS Part III for both OFF and ON states, starting from 12 and 24 months, respectively, and continuing to increase up to the 4-year time point. This finding suggests that, once established, the effect of prasinezumab may have persisted, at least for the duration of the washout period, and argues against a symptomatic effect of prasinezumab. Another important point to consider is the definition of OFF. Participants in PASADENA, if they were on levodopa, were asked not to take any levodopa from the previous night, and therefore potentially had a longer washout period than PPMI participants who were instructed not to take any levodopa for only 6 h before the planned MDS–UPDRS Part III OFF assessment. However, this does not change the interpretation or the ability to use the PPMI as an external control cohort for the present study. Indeed, the fact that participants in the PPMI had a shorter minimum washout of symptomatic treatment (6 h compared with ‘overnight’) would actually suggest that the separation that we observed between PASADENA and PPMI participants in MDS–UPDRS Part III in OFF is more likely to be underestimated than overestimated. Taken together, our results suggest that prasinezumab may slow motor progression and functional decline over the long term in early-stage PD.

We identified several limitations in this exploratory analysis. Some of these are related to potential differences between the external observational PPMI cohort and PASADENA participants. First, although the PASADENA and PPMI cohorts are comparable, there is a potential calendar time bias. The PASADENA study started in 2017, and the participants’ assessments up to the year 4 data cut snapshot occurred in the third quarter of 2023 whereas the initial PPMI cohort was enrolled between 2011 and 2018. Despite a gap of 6 years on initiation, there is a large overlap between the studies. Second, the PPMI study was conducted in North America, Europe, Israel and Australia, and PASADENA is being conducted in North America and Europe. This difference in regional bias is considered low. Third, selection bias, which refers to the enrollment of people in clinical trials that are different from those in clinical practice, is limited because PASADENA has inclusion and exclusion criteria similar to those of the PPMI study. Fourth, participants in the OLE of PASADENA and the PPMI cohort may also differ due to clinic visit frequency. The PASADENA study required a visit to the clinic every month to receive an infusion, while in the PPMI study participants visited their clinic approximately every 3 months for the first year, after which visits occurred every 6 months. Fifth, due to a lack of placebo arm in the OLE, a potential placebo effect cannot be fully ruled out. Sixth, the retrospective nature of defining the external comparator arm, based on secondary data use and applying PASADENA inclusion criteria to the PPMI cohort, introduces potential for selection bias. Seventh, although the PASADENA inclusion criteria were retrospectively applied to the PPMI study to create a comparable cohort, not all criteria were exactly the same. For example, PASADENA also included participants on stable doses of MAO-B inhibitors at baseline whereas all participants in the PPMI study were required to be untreated. However, as a sensitivity analysis of the MDS–UPDRS Parts II and III, the PPMI cohort was compared with PASADENA participants who were treatment naive at baseline, showing consistent results. In early-stage PD, clinical trials longer than 3 years would be needed to detect meaningful changes in disease progression^10^, and maintaining PD patients on a placebo arm for such a long time would be ethically challenging and probably limit participation. Thus long-term, open-label studies using comparisons with data obtained from observational cohorts offer a valuable alternative to traditional randomized controlled trials. Nonetheless, comparator arms from observational cohorts have limitations regarding potential selection bias, confounding factors (for example, diet and exercise) and data quality issues. In the present study, some of these potential limitations are mitigated by using the PPMI cohort.

Our findings need confirmation in another long-term trial. The PADOVA study (NCT04777331) is an ongoing Phase IIb, multicenter, randomized, double-blind, placebo-controlled trial evaluating the efficacy and safety of intravenous prasinezumab versus placebo in participants with early-stage PD and who are on stable symptomatic PD medication (for at least 6 months, with stable doses for 3 months before baseline). The double-blind part of the PADOVA study is followed by an OLE, which, like the present study, will address the long-term effects of prasinezumab treatment.

In conclusion, the effect of prasinezumab on slowing motor progression in PD may be sustained long term. The PASADENA OLE is continuing.

## Methods

### Ethics statement

In PASADENA, the protocol and recruitment materials used were approved by the institutional review boards or ethics committees at each study site^4^. The trial was conducted according to the principles of the Declaration of Helsinki and Good Clinical Practice guidelines. All participants provided written informed consent before participation and for secondary data use^4^. Similarly, the PPMI study was conducted in accordance with the Declaration of Helsinki and Good Clinical Practice guidelines following approval by the local ethics committees of the participating sites, and all participants provided informed consent^5^.

### Study design

Data from the PASADENA and PPMI studies were used for this exploratory analysis, and this external comparator arm was defined retrospectively based on secondary data use. PASADENA (ClinicalTrials.gov identifier no. NCT03100149) is a randomized controlled trial evaluating prasinezumab in participants with early-stage PD, details of which have been published previously^4,22^. It is being conducted at 57 sites in Austria, France, Germany, Spain and the United States^4^. A total of 316 participants with early-stage PD (diagnosis ≤2 years at screening, H&Y stages 1–2) were randomized to receive either intravenous prasinezumab (1,500 or 4,500 mg) every 4 weeks for 104 weeks during Parts 1 and 2 of the study (early-start group) or placebo for 52 weeks during Part 1, followed by prasinezumab (1,500 or 4,500 mg) for 52 weeks in Part 2 (delayed-start group). Following a minimum washout period of 3 months, participants could enter a 5-year OLE, receiving prasinezumab 1,500 mg every 4 weeks (Part 3). All PASADENA participants (*n* = 271) were considered in the analysis, regardless of change in symptomatic therapy. A total of 397 untreated individuals were enrolled in the PPMI sporadic PD cohort from July 2010 until May 2013, at 24 sites in the United States, Europe and Australia^5^. The timeframe for the current analysis is presented in Fig. 1. Covariate balancing was performed at baseline (‘Data analysis’) to provide a cohort that resembled the PASADENA cohort, and endpoints were analyzed from baseline over 4 years.

### Study population

In PASADENA, key inclusion criteria included idiopathic PD with bradykinesia and one of the other cardinal signs of PD (resting tremor, rigidity) and no other known or suspected cause of PD; age 40‒80 years; dopamine transporter imaging with DaT–SPECT consistent with PD; diagnosis of PD for 2 years or less at screening; modified H&Y stage 1 or 2; and either treatment naive or on a stable dose of an MAO-B inhibitor for at least 90 days at baseline. Key exclusion criteria included medical history indicating a Parkinson syndrome other than idiopathic PD; known carriers of certain familial PD genes (*Parkin*, *PINK1*, *DJ1*); mini mental state examination ≤25; use of catechol-*O*-methyltransferase inhibitors, amantadine, anticholinergics or dopaminergic medication for more than a total of 60 days or within 60 days of baseline; and previous participation in any prasinezumab study^4,22^.

In the PPMI study, key inclusion criteria for those with PD included age ≥30 years; DaT–SPECT or vesicular monoamine transporter (VMAT-2) imaging (Australia only) consistent with PD; untreated with PD medications (levodopa, dopamine agonists, MAO-B inhibitors or amantadine) within 2 years of diagnosis; H&Y stage 1 or 2; and the presence of either at least two of resting tremor, bradykinesia or rigidity (must have either resting tremor or bradykinesia) or a single asymmetric resting tremor or asymmetric bradykinesia^5^. Key exclusion criteria for PD participants included a clinical diagnosis of dementia or the taking of PD medications within 60 days of baseline or for >60 days in total^5^. For the current analysis, PASADENA inclusion criteria were applied to the PPMI study (age 40‒80 years and the presence of two motor features of which one is bradykinesia and the second either resting tremor or rigidity). Follow-up assessments were performed at 3-month intervals during the first year of participation, and every 6 months thereafter, up to 11 years after inclusion.

Sex (male versus female) was considered in the study design as a baseline characteristic and was included as a variable in the covariate adjustment (see ‘Data analysis’ section below). Sex was self-reported by participants. Information on gender was not collected.

### Data sample

**Extended Data Fig. 4:**
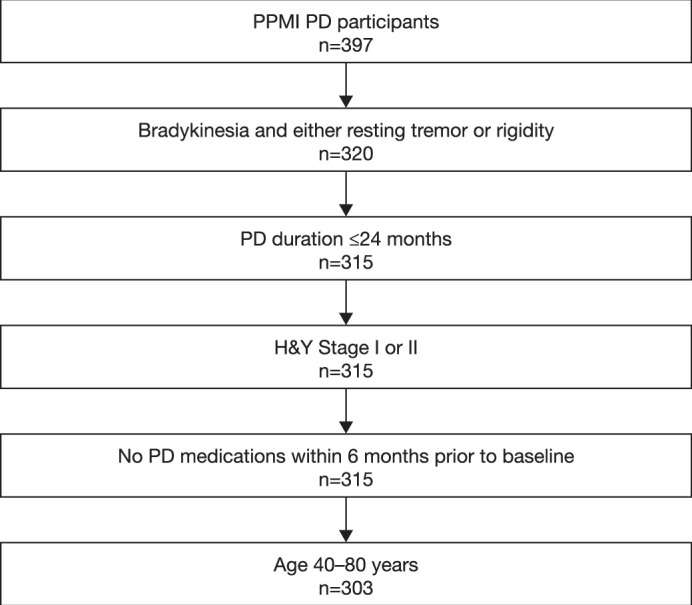
Attrition of PPMI sample. H&Y, Hoehn and Yahr; PD, Parkinson’s disease; PPMI, Parkinson’s Progression Markers Initiative.

In PASADENA, a total of 271 of 316 (85.8%) participants rolled over into the 5-year OLE after an average washout period of 7.4 months and median of 6.5 months (early-start group, *n* = 177 of 211; delayed-start group, *n* = 94 of 105). The clinical data cutoff representing a minimum of 4 years of follow-up occurred in July 2023, with a snapshot taken on 2 October 2023. The PPMI study is an observational study sponsored by The Michael J. Fox Foundation, launched in 2010 to identify biomarkers of PD onset and progression, and enrolling individuals with early-stage PD in 12 countries, in partnership with more than 30 biotech and pharmaceutical, nonprofit and private funders. We elected to use the PPMI observational study as our external comparator for PD progression because it is contemporary with PASADENA and runs in similar clinical sites. We undertook several steps to include only individuals in PPMI who closely matched the PASADENA OLE participants. We also developed a disease-modeling quantitative approach to complement the external comparator analysis. From the August 2021 version of the Analytic Dataset, the original PPMI population included 397 PD participants. Following application of the PASADENA inclusion criteria included in this analysis, 303 PD participants from the PPMI cohort were included (see Extended Data Fig. 4 for details of attrition).

For all participants, any dosages of reported symptomatic PD treatments were converted to LEDD using the methods described by Jost et al.^23^ and Tomlinson et al.^24^.

### Study endpoints

Outcomes were compared among the PASADENA early-start group, PASADENA delayed-start group and external comparator PPMI cohort.

The primary endpoints of this analysis were: change from baseline to year 4 in the severity of motor progression (irrespective of starting symptomatic treatment) as measured by change in MDS–UPDRS Parts II and III in both ON and OFF state; motor subscores (bradykinesia, rigidity, resting tremor); axial signs; and tremor motor severity subscore and nontremor motor severity subscore in PASADENA 4-year OLE participants compared with the propensity score-weighted PPMI cohort.

The secondary and exploratory endpoints were: change from baseline to year 4 in LEDD; MDS–UPDRS Part I sleep-related subscores (that is, items 7 and 8 for sleep and item 13 for fatigue); severity of motor complications as measured by MDS–UPDRS Part IV and DaT–SPECT in the bilateral putamen and bilateral caudate (secondary); and the odds of having H&Y ≥3 versus <3 (exploratory) in PASADENA 4-year OLE participants compared with the PPMI cohort.

DaT–SPECT analysis was conducted as previously described^4,5^.

### Data analysis

Data were collected using eCRF Medidata Classic Rave 2021.1.2 (Copyright 1999–2021 Medidata Solutions, Inc.). PPMI data were accessed through the Amazon Web Services Apollo platform, and PASADENA study data from Entimo’s Integrated Clinical Environment (entimICE) Framework v.2.4.30800. All analyses were performed using SAS software v.9.04 and R v.4.0.3. The statistical software R (R Core Team 2020, v.4.2.2) was used to combine the sources of data, construct the cohort, derive analysis variables and analyze data.

Two independent data analysis approaches were implemented: one based on propensity score and the other on disease modeling.

### Propensity score

A propensity score is defined as the conditional probability of treatment assignment based on observed baseline covariates, and a propensity score-adjusted analysis provides a technique to control for confounding bias and ensure comparability in observational studies^7,8^. Potential confounders as baseline characteristics were then considered, including age, sex, education, MDS–UPDRS Part III score in OFF state, modified H&Y stage, DaT–SPECT in the bilateral putamen, Montreal Cognitive Assessment score and time since diagnosis of PD. The propensity score was estimated using a logistic regression model where group assignment (PPMI versus PASADENA) was regressed on the final set of covariates. The propensity score was used to balance baseline characteristics of the PPMI cohort to resemble more closely those of the PASADENA cohort, by employing inverse probability of treatment weighting. Each participant in PASADENA was given an equal weight of 1, whereas participants in the PPMI arm were given weights calculated as propensity score/(1 − propensity score). The estimation obtained with this weighting is the average treatment effect in the treated, a term from causal inference that refers to the treatment effect for individuals who received the treatment. By doing so, a pseudopopulation was created via weighting, in which baseline covariates are balanced (that is, independent of the treatment assignment). Balancing between the PASADENA and PPMI cohorts was assessed by evaluation of SMD, and a baseline characteristic was considered balanced if its SMD following weighting was ≤0.2 (ref. ^25^). This process was repeated iteratively until the desired balance was achieved. Weights were included in the outcome models.

A mixed model for repeated measures (MMRM) was used for the longitudinal endpoints including covariates: age, sex, education, bilateral putamen at baseline, the visit (as a categorical factor), a group-by-visit interaction and the baseline endpoint. Within each participant, the model incorporates an unstructured variance–covariance matrix for the random error terms. Adjusted mean differences were extracted from the MMRM and the estimated pooled treatment effects for the overall period were summarized as the difference between the two treatment arms (PASADENA versus PPMI). Analysis of covariance was used as the primary analysis for change in DaT–SPECT areas, including the same covariates as for MMRM. A MMRM was also performed post hoc.

The validity of this methodology was confirmed by ensuring the comparability of baseline characteristics and progression over 52 weeks between the PASADENA placebo group and the weighted PPMI cohort; results were also in line with published literature^9^.

A logistic model was fitted to the number of participants with H&Y ≥3 versus <3 at year 4. Subjects who withdrew from the study up to year 4 were excluded. Missing values were considered events (26 records in PPMI, 12 in PASADENA). Due to the low number of events in PASADENA, the two arms were combined. A logistic model was fitted to the number of participants with H&Y ≥3 versus <3 at year 4, including the following covariates: age, sex, education, H&Y stage at baseline and arm.

The schedule of assessments was planned for a visit every 2 months during the first 2 years and then every 3 months, during the OLE, for participants in PASADENA while in PPMI it was every 3 months for the first year then every 6 months, with the main assessments collected yearly. Hence, we used data from the yearly visits in PASADENA (8 weeks) for this comparison to match the PPMI arm for the longitudinal endpoints.

MDS–UPDRS Part IV, which measures motor complications after the start of levodopa, was explored in two ways. Firstly, it was analyzed as a continuous endpoint including participants in the PPMI cohort or those taking prasinezumab and who started levodopa during the first 3 years. The index date was defined as the annual visit after which a participant started levodopa treatment. Cohorts were balanced at the index date on age, sex, education, MDS–UPDRS Part III ON, Part IV, H&Y, PD diagnosis, LEDD value on the day of MDS–UPDRS Part III ON assessment, starting of MAO-Bi (yes/no) and starting of dopamine agonist (yes/no). MMRM was used to model the change from index date up to year 4 with the following covariates: LEDD value on the day of MDS–UPDRS Part III ON assessment, months in the study before starting levodopa, months on prasinezumab, age, sex, education, catechol-*O*-methyltransferase-inhibitor initiation (yes/no) and amantadine initiation (yes/no).

Secondly, MDS–UPDRS Part IV was analyzed as a dichotomous endpoint including participants who had been on levodopa for at least 2 years at the end of year 4. A logistic model was fitted to the number of participants with MDS–UPDRS Part IV of ≥1 versus <1 at year 4 if at least 20% of events per arm were observed, including the covariates previously defined.

### Disease modeling

Disease modeling is a quantitative approach used to predict the progression of an endpoint or measure of efficacy as a function of a population’s characteristics. As such, it has been recognized as a key approach in supporting the development of effective therapeutic strategies^26^. We recently developed disease models of MDS–UPDRS Parts III and II based on PPMI data^10^. The models capture the natural progression of PD and the effect of symptomatic treatments on progression following a population approach^27^. The effect of symptomatic treatments was modeled by means of pharmacodynamic relationships using the LEDD^23,24^ as input of the models. Disease modeling complements the propensity score approach because it continuously balances the usage of LEDD at the subject level throughout the course of PD.

**Extended Data Fig. 5:**
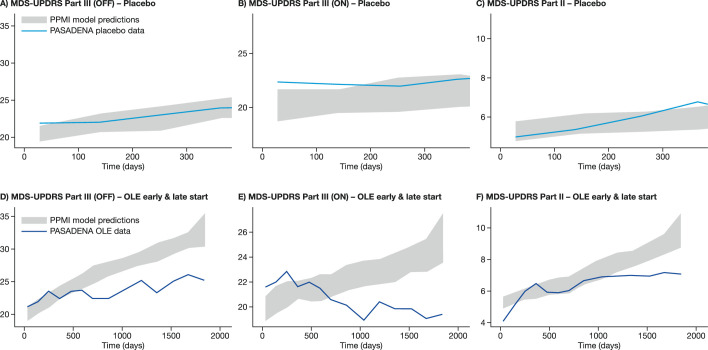
Evaluation of the PPMI-based disease progression models with PASADENA data from the placebo group, and comparison of PPMI-based disease model prediction and PASADENA data when merging early- and delayed-start groups. (**a**) MDS-UPDRS Part III OFF; (**b**) MDS-UPDRS Part III ON; (**c**) MDS-UPDRS Part II; (**d**) MDS-UPDRS Part III OFF and OLE; (**e**) MDS-UPDRS Part III ON and OLE; and (**f**) MDS-UPDRS Part II and OLE. Over that year, 46% and 48% of the PASADENA data fall within the predicted PPMI median for MDS-UPDRS Part III OFF and Part II, respectively. For MDS-UPDRS Part III ON, 42% of the PASADENA data fall within the PPMI median, which was considered too low to keep the model for further comparison. Note that the figures for MDS-UPDRS Part III ON are only shown for transparency and to illustrate the challenges of successfully reproducing placebo data. PPMI model predictions are represented by the shaded area, while PASADENA placebo and OLE data are shown as light and dark blue lines, respectively. MDS-UPDRS, Movement Disorder Society-sponsored revision of the Unified Parkinson’s Disease Rating Scale; OLE, open-label extension; PASADENA, Phase II trial of Anti alpha-Synuclein AntiboDy in Early ParkiNson's diseAse; PPMI, Parkinson’s Progression Markers Initiative.

The models were evaluated by comparison of predictions with PASADENA placebo data. For this, simulations of the models using PPMI-inferred parameters at a population level, and LEDD dosing regimen as used in the PASADENA study, were performed to form a PASADENA external comparator arm. No additional parameters were considered for matching the two cohorts beyond this balancing for LEDD usage. This prediction matched the actual data from the control group of PASADENA for MDS–UPDRS Part III OFF and Part II, but not for Part III ON. Indeed, over that year, 46 and 48% of the PASADENA data were below the predicted PPMI median for MDS–UPDRS Part III OFF and Part II, respectively. These numbers were considered satisfactory given the theoretical expectation of 50%. However, for MDS–UPDRS Part III ON, a total of 42% was below the predicted median. Based on this, and supported by the visual predictive check (Extended Data Fig. 5), we decided not to use the PPMI-based Part III ON disease model for further comparison with PASADENA. Predictions of MDS–UPDRS Part III OFF and Part II from the virtual control arm were then compared with empirical data from the PASADENA treatment groups. This comparison was performed both qualitatively and quantitatively through prediction-corrected visual and numerical predictive checks^28^.

### Reporting summary

Further information on research design is available in the Nature Portfolio Reporting Summary linked to this article.

## Online content

Any methods, additional references, Nature Portfolio reporting summaries, source data, extended data, supplementary information, acknowledgements, peer review information; details of author contributions and competing interests; and statements of data and code availability are available at 10.1038/s41591-024-03270-6.

## Supplementary information

Supplementary Information Supplementary Results and Figs. 1 and 2; full list of members of the PASADENA investigators and Prasinezumab Study Group.

Reporting Summary

## Figures and Tables

**Extended Data Table 1:**
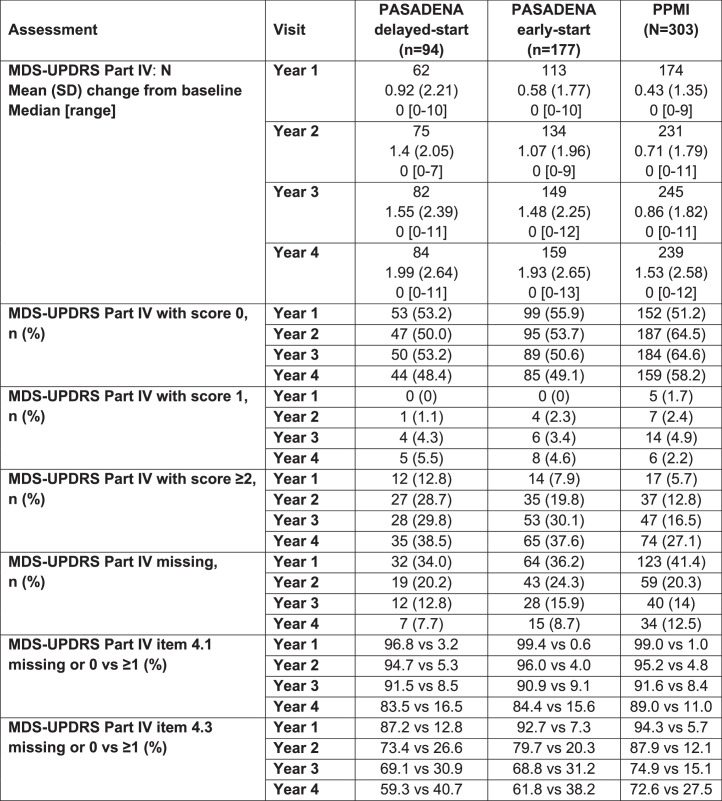
MDS-UPDRS Part IV – descriptive summary statistics

MDS-UPDRS, Movement Disorder Society-sponsored revision of the Unified Parkinson’s Disease Rating Scale; SD, standard deviation; PASADENA, Phase II trial of Anti alpha-Synuclein AntiboDy in Early ParkiNson's diseAse; PPMI, Parkinson’s Progression Markers Initiative.

**Extended Data Table 2:**
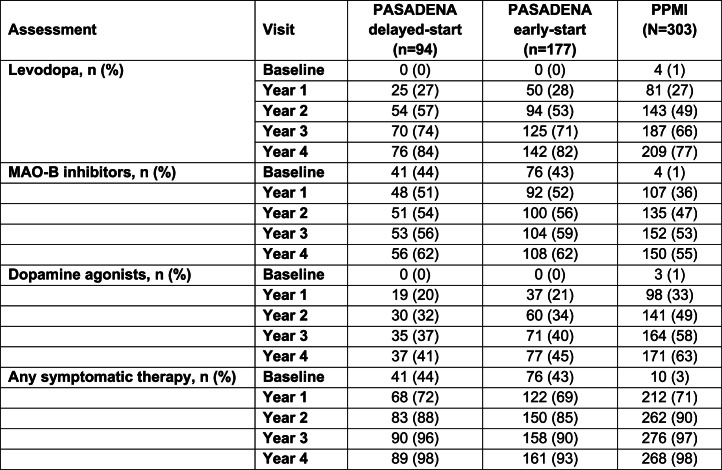
Starting of symptomatic therapies – descriptive summary statistics

MAO-B, monoamine oxidase type B; PASADENA, Phase II trial of Anti alpha-Synuclein AntiboDy in Early ParkiNson's diseAse; PPMI, Parkinson’s Progression Markers Initiative.

## Data Availability

For PASADENA data: Qualified researchers may request access to individual patient-level data through the clinical study data request platform (https://vivli.org/). Further details on Roche’s criteria for eligible studies are available at https://vivli.org/members/ourmembers/. For further details on Roche’s Global Policy on the Sharing of Clinical Information and how to request access to related clinical study documents, see https://www.roche.com/research_and_development/who_we_are_how_we_work/clinical_trials/our_commitment_to_data_sharing.htm. PPMI data used in the preparation of this article were obtained (in September 2022) from the PPMI database (www.ppmi-info.org/access-dataspecimens/download-data), RRID:SCR 006431. For up-to-date information on the study, visit www.ppmi-info.org The datasets generated and analyzed that support the conclusions of this study are available via figshare at 10.6084/m9.figshare.25541221 (ref. ^29^).
