# Supplementary material for: Sustained effect of prasinezumab on Parkinson’s disease motor progression in the open-label extension of the PASADENA trial
Source: Nat Med. 2024 Oct 8;30(12):3669–75. doi: 10.1038/s41591-024-03270-6 (PMC11645263; doi:10.1038/s41591-024-03270-6)
Supplement: Supplementary file 1 — Supplementary Results and Figs. 1 and 2; full list of members of the PASADENA investigators and Prasinezumab Study Group. [file 41591_2024_3270_MOESM1_ESM.pdf]

nature medicine

Supplementary information https://doi.org/10.1038/s41591-024-03270-6

Sustained effect of prasinezumab on Parkinson's disease motor progression in the open-label extension of the PASADENA trial

In the format provided by the authors and unedited

Supplementary Information

Contents

Supplementary Results **1**
Sensitivity analysis in treatment-naïve participants 1
Additional DaT-SPECT results.. 1
Rigidity subscore 2
Resting tremor subscore 2
Bradykinesia subscore 2
Axial signs subscore.. 2
Tremor motor severity subscore – ON-state 3
Tremor motor severity subscore – OFF-state 3
Non-tremor subscale – ON-state 3
Supplementary Figure 1. Adjusted mean change from in treatment-naïve participants for (A) MDS-UPDRS Part III motor in OFF-state, (B) MDS-UPDRS Part III motor in ON-state, and (C) MDS-UPDRS Part II. **5**
Supplementary Figure 2. Adjusted mean change from in MDS-UPDRS Part III motor subscores (all in OFF state), and in combination with Part II for tremor and non-tremor. **6**
A full list of the members of the PASADENA Investigators and Prasinezumab Study Group **8**
PASADENA Investigators... 8
Prasinezumab Study Group.. 12

Supplementary Results

Sensitivity analysis in treatment-naïve participants

The treatment-naïve participants in the PASADENA delayed- (n=53) and early-start (n=101) groups showed a slower decline (a smaller increase in score) in MDS-UPDRS Part III scores in the OFF-state (-50% for the delayed-start group and -66% for the early-start group, (**Supplementary Figure 1A**), MDS-UPDRS Part III in the ON-state (-116% for the delayed-start group and -115% for the early-start group, **Supplementary Figure 1B**), and MDS-UPDRS Part II (-46% for the delayed-start group and -33% for the early-start group, **Supplementary Figure 1C**) than did the PPMI external comparator (n=303).

Additional DaT-SPECT results

As a descriptive analysis, the PASADENA delayed- and early-start groups showed a mean progression after 4 years of 0.63 (80% CI, 0.6 to 0.65) and 0.63 (80% CI, 0.61 to 0.65) points, respectively, while the PPMI cohort showed a mean progression of 0.55 (80% CI, 0.53 to 0.56) points in the putamen. In the caudate, the PASADENA delayed- and early-start groups showed a mean progression after 4 years of 1.18 (80% CI, 1.13 to 1.23) and 1.19 (80% CI, 1.15 to 1.23) points, respectively, while the PPMI cohort showed a mean progression of 1.45 (80% CI, 1.45 to 1.49) points.

The annual decline in putamen at year 1, 2 and 4 in PASADENA was -6.66, -10.55 and -9.02 (delayed-start) and -10.33, -8.90 and -8.41 (early-start) percent, while for the PPMI cohort, it was -16.70, -6.45 and -6.53 percent, respectively. The annual decline in caudate at year 1, 2 and 4 in PASADENA was -6.42, -9.37 and -8.85 (delayed-start) and -8.84, -8.75 and -8.53 (early-start) percent, while for the PPMI cohort, it was 10.11 -7.63, and -5.07 percent, respectively.

Rigidity subscore

The PASADENA delayed- and early-start groups both showed lower MDS-UPDRS Part III rigidity subscore progression in OFF-state in comparison with the PPMI cohort after 4 years, with -39% relative difference (mean [80% CI], -0.77 [-1.25 to -0.29] points) for the delayed-start group and -71% relative difference (mean [80% CI], -1.38 [-1.77 to -0.98] points) for the early-start group (**Supplementary Figure S2A**).

Resting tremor subscore

The PASADENA delayed- and early-start groups both showed lower MDS-UPDRS Part III resting tremor subscore progression in OFF-state in comparison with the PPMI cohort after 4 years, with -34% relative difference (mean [80% CI], -0.47 [-0.89 to -0.06] points) for the delayed-start group and -50% relative difference (mean [80% CI], -0.69 [-1.03 to -0.35] points) for the early-start group (**Supplementary Figure S2B**).

Bradykinesia subscore

The PASADENA delayed- and early-start groups both showed lower MDS-UPDRS Part III bradykinesia subscore progression in OFF-state in comparison with the PPMI cohort after 4 years, with -62% relative difference (mean [80% CI], -3.67 [-4.70 to -2.65] points) for the delayed-start group and -67% relative difference (mean [80% CI], -3.98 [-4.83 to -3.13] points) for the early-start group (**Supplementary Figure S2C**).

Axial signs subscore

Axial symptoms comprise MDS-UPDRS Part III items 10, 11 and 12. The PASADENA delayed- and early-start groups both showed lower MDS-UPDRS Part III axial signs subscore progression in OFF-state in comparison with the PPMI cohort after 4 years, with -67% relative difference (mean [80% CI], -0.47 [-0.71 to -0.23] points) for the delayed-start

group and -64% relative difference (mean [80% CI], -0.45 [-0.65 to -0.25] points) for the early-start group (**Supplementary Figure S2D**).

Tremor motor severity subscore – ON-state

The tremor motor severity subscore includes item 10 from MDS-UPDRS Part II and items 15, 16, 17 and 18 from MDS-UPDRS Part III. The PASADENA delayed- and early-start groups both showed lower MDS-UPDRS Part III tremor subscore progression in ON-state in comparison with the PPMI cohort after 4 years, with -19% relative difference (mean [80% CI], 0.3 [-0.26 to 0.86] points) for the delayed-start group and -35% relative difference (mean [80% CI], 0.55 [0.1 to 1.01] points) for the early-start group (**Supplementary Figure S2E**).

Tremor motor severity subscore – OFF-state

The PASADENA delayed- and early-start groups both showed lower MDS-UPDRS Part III tremor subscore progression in OFF-state in comparison with the PPMI cohort after 4 years, with 400% relative difference (mean [80% CI], 0.64 [-0.02 to 1.29] points) for the delayed-start group and 288% relative difference (mean [80% CI], 0.46 [-0.06 to 0.98] points) for the early-start group (**Supplementary Figure S2F**).

Non-tremor subscale – ON-state

The non-tremor motor severity subscore includes items 1–9 and 11–13 from MDS-UPDRS Part II, and items 1–14 from MDS UPDRS Part III. The PASADENA delayed- and early-start groups both showed lower MDS-UPDRS Part III non-tremor subscore progression in ON-state in comparison with the PPMI cohort after 4 years, with -53% relative difference (mean [80% CI], -2.97 [-4.97 to -0.98] points) for the delayed-start group and -66% relative difference (mean [80% CI], -3.75 [-5.38 to -2.12] points) for the early-start group (**Supplementary Figure S2G**).

Non-tremor subscale – OFF-state

The PASADENA delayed- and early-start groups both showed lower MDS-UPDRS Part III non-tremor subscore progression in OFF-state in comparison with the PPMI cohort after 4 years, with 9% relative difference (mean [80% CI], 0.47 [-1.78 to 2.73] points) for the delayed-start group and -2% relative difference (mean [80% CI], -0.08 [-1.9 to 1.74] points) for the early-start group (**Supplementary Figure S2H**).

Supplementary Figure 1. Adjusted mean change from in treatment-naïve participants for (A) MDS-UPDRS Part III motor in OFF-state, (B) MDS-UPDRS Part III motor in ON-state, and (C) MDS-UPDRS Part II. Error bars represent 80% confidence intervals. MDS-UPDRS, Movement Disorder Society-sponsored revision of the Unified Parkinson's Disease Rating Scale; PPMI, Parkinson's Progression Markers Initiative.

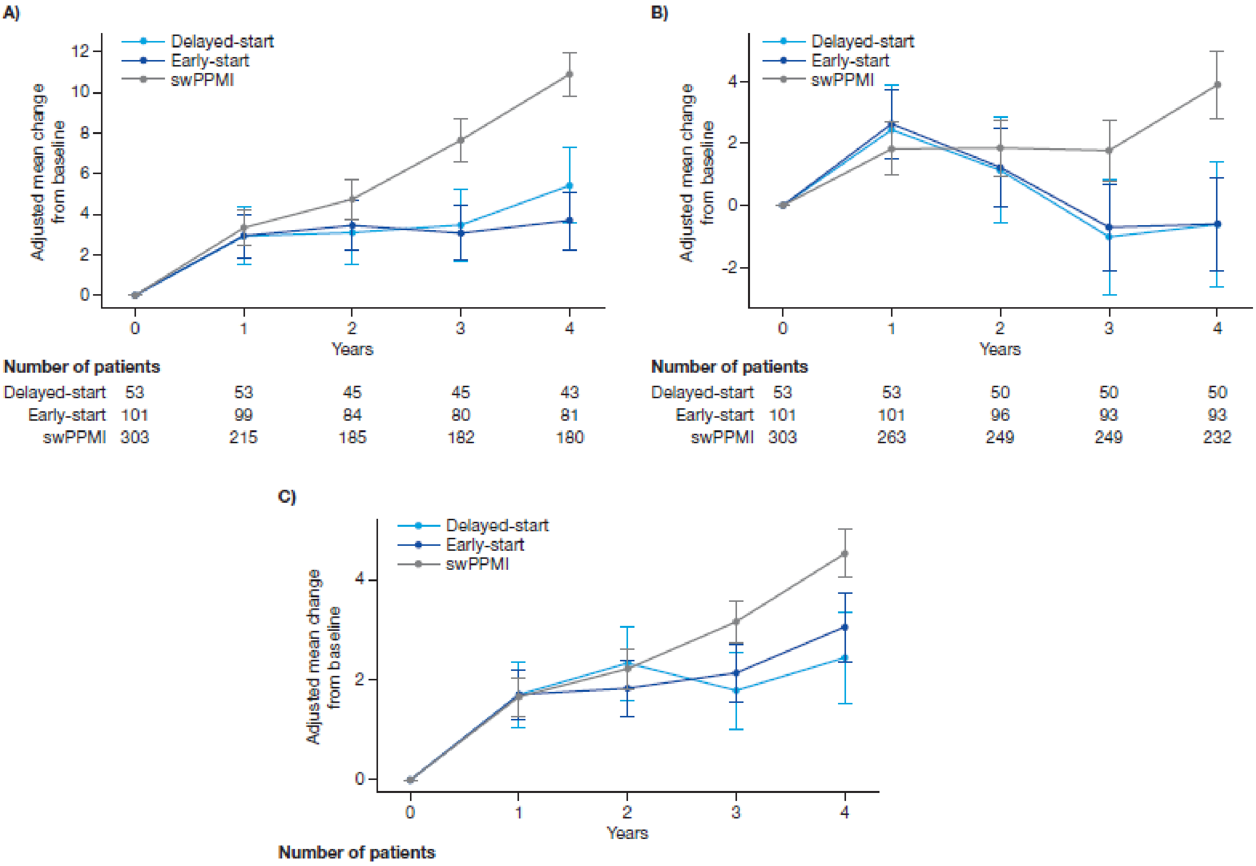

A) Number of patients

	0	1	2	3	4
Delayed-start	53	53	45	45	43
Early-start	101	99	84	80	81
swPPMI	303	215	185	182	180

B) Number of patients

	0	1	2	3	4
Delayed-start	53	53	50	50	50
Early-start	101	101	96	93	93
swPPMI	303	263	249	249	232

C) Number of patients

	0	1	2	3	4
Delayed-start	53	53	52	50	51
Early-start	101	101	100	93	93
swPPMI	303	268	262	265	248

Supplementary Figure 2. Adjusted mean change from in MDS-UPDRS Part III motor subscores (all in OFF state), and in combination with Part II for tremor and non-tremor. Error bars represent 80% confidence intervals. MDS-UPDRS, Movement Disorder Society-sponsored revision of the Unified Parkinson's Disease Rating Scale; PPMI, Parkinson's Progression Markers Initiative.

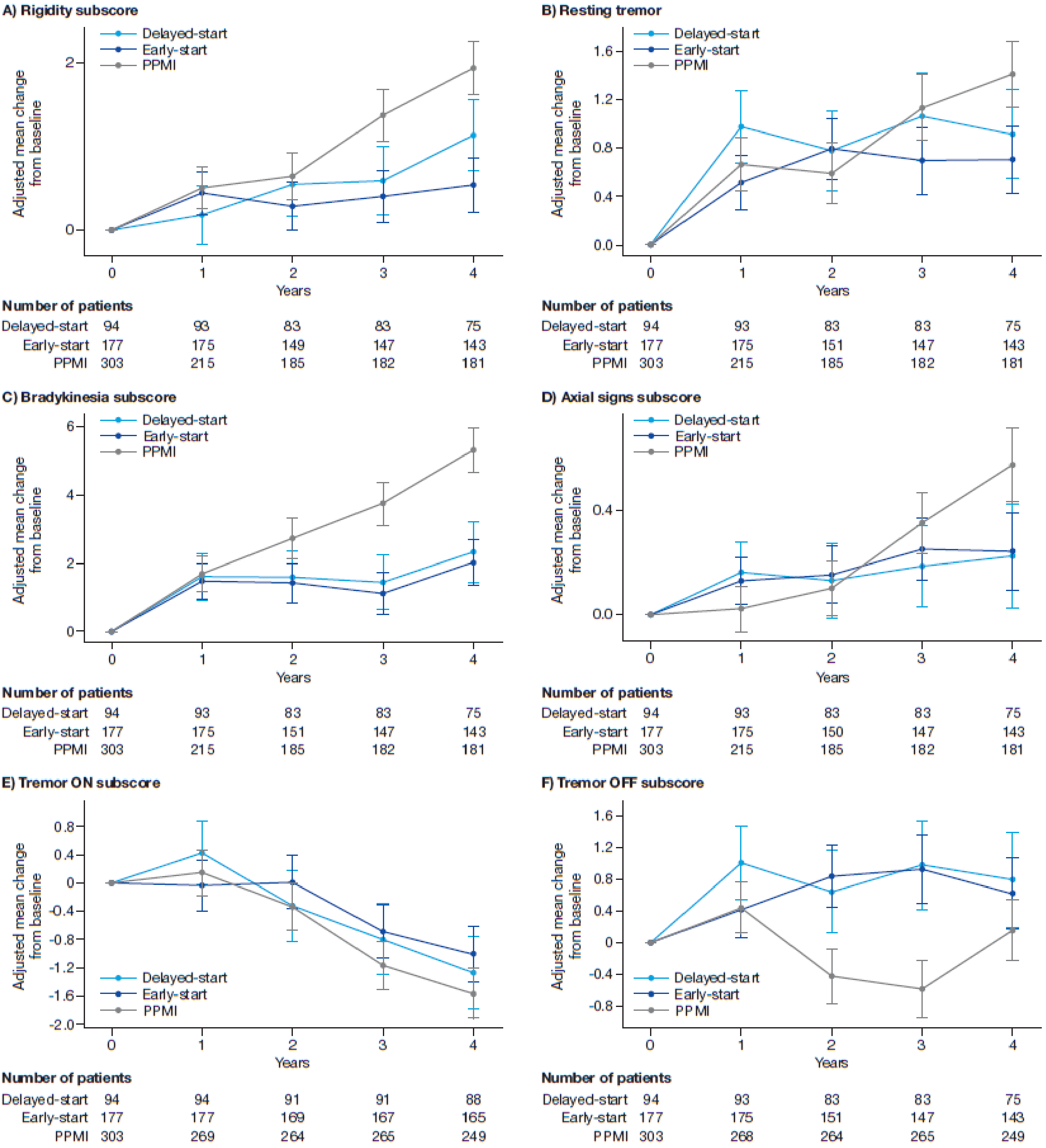

G) Non-tremor ON subscore

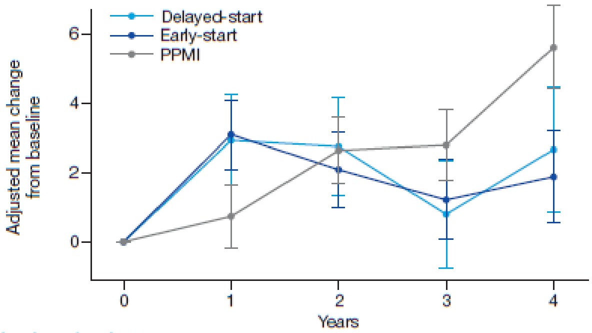

Number of patients

	0	1	2	3	4
Delayed-start	94	94	91	91	88
Early-start	177	177	169	167	165
PPMI	303	269	264	265	249

H) Non-tremor OFF subscore

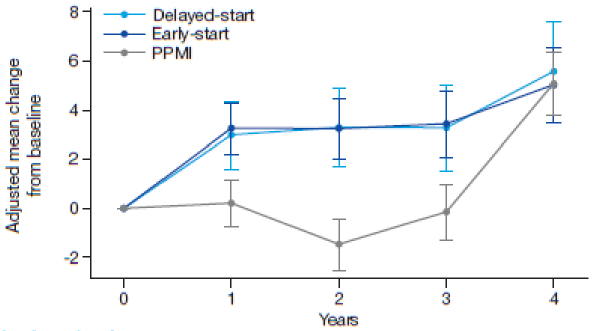

Number of patients

	0	1	2	3	4
Delayed-start	94	93	83	83	75
Early-start	177	175	151	147	143
PPMI	303	268	264	265	249

A full list of the members of the PASADENA Investigators and Prasinezumab Study Group

PASADENA Investigators

Claudia Altendorf[1], Chareyna Anandan[2], Giulia Andrews[3], Solène Ansquer[4], Raphaele Arrouasse[5], Sana Aslam[3], Jean-Philippe Azula[6], Jeanette Baker[7], Ernest Balaguer Martinez[8], Shadi Barbu[3], Kara Bardram[9], Danny Bega[10], Helena Bejr-Kasem Marco[11], Isabelle Benatru[4], Eve Benchetrit[6], Felix Bernhard[12], Amit Besharat[13], Sagari Bette[14], Amelie Bichon[15], Andrew Billnitzer[2], Sophie Blondeau[4], Thomas Boraud[16], Freiderike Borngräber[1], James Boyd[7], Kathrin Brockmann[17], Matthew Brodsky[18], Ethan Brown[19], Christof Bruecke[1], Fabienne Calvas[20], Monica Canelo[21], Federico Carbone[22], Claire Carroll[23], Laura Casado Fernandez[24], Catherine Casse-Parrot[6], Anna Castrioto[15], Helene Catala[20], Justine Chan[3], Samia Cheriet[20], Anthony Ciabarra[25], Joseph Classen[26], Juliana Coleman[27], Robert Coleman[27], Yaroslau Compta[28], Jean-Christophe Corvol[29], Mariana Cosgaya[28], Nabila Dahodwala[30], Philippe Damier[31], Elodie David[32], Thomas Davis[33], Marissa Dean[34], Berengere Debilly[35], Janell DeGiorgio[36], Andres Deik[30], Laure Delaby[35], Marie-Helene Delfini[6], Pascal Derkinderen[31], Philipp Derost[35], Maria de Toledo[24], Lisa Deuel[7], Ann Marie DiazHernandez[14], Cameron Dietiker[19], Karina Dimenshteyn[37], Julio Dotor[24], Franck Durif[35], Jens Ebentheuer[21], Karla Maria Eggert[38], Sara Eichau Madueño[39], Claudia Eickoff[37], Aaron Ellenbogen[9], Philipp Ellmerer[22], Ines Esparragosa Vazquez[40], Alexandre Eusebio[6], Siobhan Ewert[1], John Fang[33], Danielle Feigenbaum[13], Frederique Fluchere[6], Alexandra Foubert-Samier[16], Marie Fournier[15], Anne Fradet[4], Valerie Fraix[15], Samuel Frank[41], Franka Fries[17], Monique Gailitzky[20], Anne Gaille Corbille[31], Marisol Gallardó Pérez[42], Jose Manuel Garcia Morena[39], Carmen Gasca[43], Thomas Gasser[17], Joyce Gibbons[44], Caroline Giordana[32], Alicia Gonzalez Martinez[24], Ira Goodman[23], Arantza Gorospe[40], Marie Goubeaud[20], David Grabli[29], Mangone Graziella[29], Stephan Grimaldi[6], Jeffrey Gross[45], Raquel Guimaraes-Costa[29], Andreas Hartmann[29], Christian Hartmann[37], Travis Hassell[33], Robert Hauser[46], Antonio Hernandez[8], Jorge Hernandez-Vara[47], Günter Höglinger[48], Christian Homedes[8], Andrea

Horta[11], Jean-Luc Houeto[4], Julius Huebl[1], Jennifer Hui[13], Stuart Isaacson[14], Joseph Jankovic[2], Annette Janzen[38], Jocelyne Jiao[18], Maria Jose Marti Domenech[49], Xavier Joseph[5], Srinath Kadimi[45], Pat Kaminski[50], Silja Kannenberg[37], R. Jan Kassubek[51], Maya Katz[19], Kevin Klos[52], Shannon Klos[52], Christopher Kobet[27], Jennifer Koebert[46], Patricia Krause[1], Andrea Kuhn[1], Jaime Kulisevsky Bojarsky[53], Rajeev Kumar[36], Martin Kunz[52], Lille Kurvits[1], Kimberly Kwei[54], Simon Laganiere[41], Brice Laurens[16], Johannes Levin[48], Oren Levy[55], Peter Le Witt[51], Gurutz Linazasoro Cristobal[11], Irene Litvan[55], Karlo Lizarraga[56], Katherine Longardner[57], Rocio Lopez[39], Lydia Lopez Manzanares[24], Sara Lucas del Pozo[47], Maria Rosario Luquin Puido[40], Nijee Luthra[19], Kelly Lyons[57], Sylvia Maass[48], Gerrit Machetanz[17], Yolanda Macias[43], David Maltete[58], Jorge Uriel Manez Miro[43], Louis-Laure Mariani[29], Juan Marin[11], Kathrin Marini[22], Ana Marques[35], Gloria Marti[40], Saul Martinez[11], Wassilios Meissner[16], Sara Meoni[15], Brit Mollenhauer[21], Dunia Mon Martinez[8], Johnson Moon[25], Elena Moro[15], Peter Morrison[56], Christoph Muehlberg[26], Manpreet Multani[25], Christine Murphy[23], Anthony Nicholas[34], Rajesh Pahwa[57], Antonio Palasis[47], Heidi Pape[38], Neepa Patel[50], Prity Patel[23], Marina Peball[22], Elizabeth Peckham[59], Terry Peery[59], Jesus Perez[11], Rafael Perez Alisa Petit[39], Elmar Pinkhardt[51], Werner Poewe[22], Elsa Pomies[20], Cecile Preterre[31], Joseph Quinn[18], Olivier Rascol[20], Philippe Remy[5], Emily Reuther[27], Irene Richard[56], Benjamin Roeben[17], Jost-Julian Rumpf[26], David Russell[44], Hayet Salhi[5], Daniela Samaniego[47], Alexandra Samier-Foubert[16], Alvaro Sanchez-Ferro[43], Emmanuelle Schmitt[15], Alfons Schnitzler[37], Oliver Schorr[22], Julie Schwartzbard[60], Kerstin Schweyer[48], Klaus Seppi[22], Victoria Sergo[36], Holly Shill[3], Andrew Siderowf[30], Tanya Simuni[10], Umberto Spampinato[16], Ashok Sriram[27], Natividad Stover[34], Caroline Tanner[19], Arjun Tarakad[2], Carolyn Taylor[10], Claire Thalamus[20], Thomas Toothaker[45], Nadege Van Blercom[11], Nora Vanegas-Arrogave[54], Lydia Vela[43], Sylvian Vergnet[16], Tiphaine Vidal[35], Jonathan Vöglein[48], Ryan Walsh[3], Cheryl Waters[54], Mirko Wegschneider[26], Endy Weidinger[26], Caroline Weill[5], Gregor Wenzel[1], Tatiana Witjas[6], Isabel Wurster[17], Brenton Wright[55], Milan Zimmermann[17], Rafael Zuzuarregui[19]

1. Berlin Medical University, Neurology Clinic, Campus Charité Mitte, Berlin, Germany;
2. Baylor College of Medicine, Houston, TX, USA;
3. Barrow Neurological Institute, Phoenix, AZ, USA;
4. Poitiers University Hospital, Poitiers, France;
5. Henri-Mondor University Hospital, Créteil, France;
6. Marseille University Hospital Timone, Marseille, France;
7. University of Vermont, Larner College of Medicine, Burlington, VT, USA;
8. General University Hospital of Catalonia, Barcelona, Spain;
9. Quest Research Institute, Farmington Hills, MI, USA;
10. Northwestern University, Evanston, IL, USA;
11. Policlinica Gipuzkoa Servicio De Neurologia, Gipuzkoa, Spain;
12. Philipps University of Marburg, Neurology Clinic, Marburg, Germany;
13. University of Southern California, Keck Medical Center, Los Angeles, CA, USA;
14. Parkinson's Disease and Movement Disorders Center of Boca Raton, Boca Raton, FL, USA;
15. Grenoble Alpes University Michallon Hospital, La Tronche, France;
16. Hospital Pellegrin Bordeaux, Bordeaux, France;
17. Tubingen University Hospital, Tubingen, Germany;
18. Oregon Health & Science University, Portland, OR, USA;
19. University of California, San Francisco, CA, USA;
20. Toulouse University, Clinical Research Center, Purpan Hospital, Toulouse, France;
21. Goettingen University Medical Center, Paracelsus Elena Klinik Kassel, Goettingen, Germany;
22. Medical University of Innsbruck, Neuroradiology Clinic, Innsbruck, Austria;
23. Compass Research LLC, Orlando, FL, USA;
24. De La Princessa University Hospital, Madrid, Spain;
25. Neurology Center of North Orange County, Fullerton, CA, USA;
26. Leipzig University, Neurology Clinic and Polyclinic, Leipzig, Germany;

27. Spectrum Health Medical Group, USA;
28. Hospital Clinic Barcelona, Barcelona, Spain;
29. Sorbonne University, Pitié-Salpêtrière University Hospital, Paris, France;
30. University of Pennsylvania, Philadelphia, PA, USA;
31. Nantes University, North Laennec University Hospital, Saint-Herblain, France;
32. Nice University, Hospital Pasteur, Nice, France;
33. Vanderbilt University Medical Center, Nashville, TN, USA;
34. University of Alabama, UAB Medicine, Birmingham, AL, USA;
35. Clermont-Ferrand University Hospital Center, Site Gabriel-Montpied, Clermont-Ferrand, France;
36. Rocky Mountain Movement Disorders Center, Englewood, CO, USA;
37. Heinrich Heine University Düsseldorf, University Hospital Düsseldorf, Germany;
38. Philips University of Marburg, Marburg, Germany;
39. University of Sevilla, Virgin Macarena University Hospital, Sevilla, Spain;
40. University of Navarra, Navarra University Hospital, Department of Neurology, Navarre, Spain;
41. Beth Israel Deaconess Medical Center, Boston, MA, USA;
42. University of Barcelona, The Provincial Clinic Hospital, Barcelona, Spain;
43. University of Madrid, The Alcorcón Foundation University Hospital, Madrid, Spain;
44. Invicro, New Haven, CT, USA;
45. Associated Neurologists of Southern Connecticut, P.C., Milford, CT, USA;
46. University of South Florida, Parkinson's Disease and Movement Disorders, Tampa, FL, USA;
47. Vall d'Hebron Barcelona University Hospital, Department of Neurology, Barcelona, Spain;
48. Technical University of Munich, The Rechts der Isar Hospital, Munich, Germany;
49. Neurosciences Clinic Institute of the Barcelona Hospital Clinic, Parkinson Disease and Movement Disorders Unit, Barcelona, Spain;

50. Henry Ford Health System. Clarkston, MI, USA;

51. University of Ulm, Ulm University Hospital, Clinic for Neurology, Ulm, Germany;

52. The Movement Disorder Clinic of Oklahoma, Tulsa, OK, USA;

53. Autonomous University of Barcelona, Hospital de la Santa Creu i Sant Pau, Autoimmune Neurology Unit – Neurology Service, Barcelona, Spain;

54. Columbia University, New York, CU, USA;

55. University of California San Diego Altman Clinical and Translational Research Institute, La Jolla, CA, USA;

56. University of Rochester Medical Center, Rochester, NY, USA;

57. University of Kansas Medical Center, Kansas City, KS, USA;

58. University of Rouen Normandy Hospital, Charles Nicolle Parkinson's Disease Center, Rouen, France;

59. Central Texas Neurology Consultants, Round Rock, TX, USA;

60. Aventura Neurologic Associates, Aventura, FL, USA.

Prasinezumab Study Group

Markus Abt,[1] Atieh Bamdadian,[1] Teresa Barata,[1] Nicholas Barbet,[1] Sara Belli,[1] Frank Boess,[1] Azad Bonni,[1] Edilio Borroni,[1] Anne Boulay,[1] Markus Britschgi,[1] Valerie Cosson,[1] Christian Czech,[1] Evan Davies,[1] Dennis Deptula,[1] Cheikh Diack,[1] Rachelle Doody,[1] Juergen Dukart,[1] Giulia D'Urso,[1] Sebastian Dziadek,[1] Chris Edgar,[1] Laurent Essioux,[1] Morgan Farell,[1] Rebecca Finch,[1] Paulo Fontoura,[1] Waltraud Gruenbauer,[1] Andrea Hahn,[1] Stefan Holiga,[1] Michael Honer,[1] Shirin Jadidi,[1] Timothy Kilchenmann,[1] Thomas Kremer,[1] Thomas Kustermann,[1] Claire Landsdall,[1] Michael Lindemann,[1] Florian Lipsmeier,[1] Cecile Luzy,[1] Marianne Manchester,[1] Maddalena Marchesi,[1] Ferenc Martenyi,[2] Meret Martin-Facklam,[1] Katerina Mironova,[1] Emma Moore,[1] Annabelle Monnet,[1] Markus Niggli,[1] Tania Nikolcheva,[2] Susanne Ostrowitzki,[1] Gennaro Pagano,[1] Benedicte Passemard,[1] Agnes Poirier,[1] Anke Post,[1] Megana Prasad,[1] Nathalie Pross,[1] Benedicte Ricci,[1] Ellen Rose,[2] Daria Rukina,[1]

Christoph Sarry,[1] Marzia A. Scelsi,[1] Christine Schubert,[1] Jeff Sevigny,[1] Kaycee Sink,[1] Nima Shariati,[1] Alexander Strasak,[1] Hannah Staunton,[1] Hanno Svoboda,[1] Kirsten I. Taylor,[1] Dylan Trundell,[1] Daniel Umbricht,[1] Lynne Verselis,[1] Annamarie Vogt,[1] Ekaterina Volkova-Volkmar,[1] Cornelia Weber,[1] Silke Weber,[1] Stefano Zanigni[1]

1. F. Hoffmann-La Roche Ltd, Basel, Switzerland;

2. Prothena Corporation plc, Dublin, Ireland
