# Supplementary material for: Sustained effect of prasinezumab on Parkinson’s disease motor progression in the open-label extension of the PASADENA trial
Source: Nat Med. 2024 Oct 8;30(12):3669–75. doi: 10.1038/s41591-024-03270-6 (PMC11645263; doi:10.1038/s41591-024-03270-6)
Supplement: Supplementary file 2 — Reporting Summary [file 41591_2024_3270_MOESM2_ESM.pdf]

nature portfolio

Corresponding author(s): Gennaro Pagano

Last updated by author(s): 15.08.2024

Reporting Summary

Nature Portfolio wishes to improve the reproducibility of the work that we publish. This form provides structure for consistency and transparency in reporting. For further information on Nature Portfolio policies, see our Editorial Policies and the Editorial Policy Checklist.

Statistics

For all statistical analyses, confirm that the following items are present in the figure legend, table legend, main text, or Methods section.

n/a	Confirmed	
☐	☒	The exact sample size (*n*) for each experimental group/condition, given as a discrete number and unit of measurement
☐	☒	A statement on whether measurements were taken from distinct samples or whether the same sample was measured repeatedly
☐	☒	The statistical test(s) used AND whether they are one- or two-sided<br>*Only common tests should be described solely by name; describe more complex techniques in the Methods section.*
☐	☒	A description of all covariates tested
☒	☐	A description of any assumptions or corrections, such as tests of normality and adjustment for multiple comparisons
☐	☒	A full description of the statistical parameters including central tendency (e.g. means) or other basic estimates (e.g. regression coefficient) AND variation (e.g. standard deviation) or associated estimates of uncertainty (e.g. confidence intervals)
☒	☐	For null hypothesis testing, the test statistic (e.g. *F*, *t*, *r*) with confidence intervals, effect sizes, degrees of freedom and *P* value noted<br>*Give P values as exact values whenever suitable.*
☒	☐	For Bayesian analysis, information on the choice of priors and Markov chain Monte Carlo settings
☒	☐	For hierarchical and complex designs, identification of the appropriate level for tests and full reporting of outcomes
☐	☒	Estimates of effect sizes (e.g. Cohen's *d*, Pearson's *r*), indicating how they were calculated

Our web collection on statistics for biologists contains articles on many of the points above.

Software and code

Policy information about availability of computer code

Data collection	Data were collected using the eCRF Medidata Classic Rave® 2021.1.2 (Copyright © 1999-2021 Medidata Solutions, Inc.)
Data analysis	Data were analyzed using SAS software version 9.04 and R version 4.0.3.<br>The PPMI data were accessed through the Amazon Web Services (AWS) Apollo platform, and PASADENA study data from Entimo's Integrated Clinical Environment (entimICE®) Framework Version 2.4.30800. The statistical software R (R Core Team 2020; version 4.2.2) was used to combine the sources of data, construct the cohort, derive analysis variables, and analyze the data.

For manuscripts utilizing custom algorithms or software that are central to the research but not yet described in published literature, software must be made available to editors and reviewers. We strongly encourage code deposition in a community repository (e.g. GitHub). See the Nature Portfolio guidelines for submitting code & software for further information.

Data

Policy information about availability of data

All manuscripts must include a data availability statement. This statement should provide the following information, where applicable:

- Accession codes, unique identifiers, or web links for publicly available datasets
- A description of any restrictions on data availability
- For clinical datasets or third party data, please ensure that the statement adheres to our policy

For PASADENA data: Qualified researchers may request access to individual patient-level data through the clinical study data request platform (https://vivli.org/).

Further details on Roche's criteria for eligible studies are available here (https://vivli.org/members/ourmembers/). For further details on Roche's Global Policy on the Sharing of Clinical Information and how to request access to related clinical study documents, see here: (https://www.roche.com/research_and_development/who_we_are_how_we_work/clinical_trials/our_commitment_to_data_sharing.htm)
For PPMI data: Data used in the preparation of this article were obtained [in September 2022] from the Parkinson's Progression Markers Initiative (PPMI) database (www.ppmi-info.org/access-dataspecimens/download-data), RRID:SCR 006431. For up-to-date information on the study, visit www.ppmi-info.org.

Research involving human participants, their data, or biological material

Policy information about studies with human participants or human data. See also policy information about sex, gender (identity/presentation), and sexual orientation and race, ethnicity and racism.

Reporting on sex and gender	Sex (male vs female) was considered in the study design as a baseline characteristics and was included as a variable in the covariate adjustment. Sex was self reported by the participants. The majority of trial participants were male (PASADENA: 188/271; 69.4%; PPMI: 202/303; 66.7%). With more men than women diagnosed with Parkinson's Disease by a ratio approximately of 2:1, the proportion of male to female participants recruited in this trial is appropriate. Gender was not collected.
Reporting on race, ethnicity, or other socially relevant groupings	N/A
Population characteristics	Covariate-relevant population characteristics of the participants included: group (early start, delayed start, PPMI cohort), age at baseline (>60 / <60), sex (male/female), education (<12 years/>12 years), MAO-B inhibitor (yes/no; at year 1) and the DaT-SPECT contralateral binding ratio at baseline (at year 1).
Recruitment	PASADENA: Participants were identified for potential recruitment per site-specific recruitment plans prior to consenting to take part in this study. Any recruitment materials for participants received Institutional Review Board or Ethics Committee (IRB/EC) approval prior to use.<br><br>PPMI: PD and HC subjects of similar age and gender from 24 study sites in the US (18), Europe (5) and Australia (1) were enrolled after obtaining informed consent.
Ethics oversight	PASADENA: The trial was conducted according to the principles of the Declaration of Helsinki and Good Clinical Practice guidelines and was approved by central institutional review boards (Ethikkommission der Medizinischen Universität Innnsbruck [Austria], Comité de Protection des Personnes [CPP] Ouest IV [France], Ethikkommission der LÄK Hessen [Germany], CEIm Hospital Universitari Vall d'Hebron [Spain], Copernicus Group Independent Review Board [US] and Western Institutional Review Board [US]) or ethics committees at each trial site (Ethikkommission der Universität Leipzig Geschäftsstelle der Ethikkommission an der medizinischen Fakultät der Universität Leipzig [Germany], Ethikkommission der Fakultät für Medizin der Technischen Universität München [Germany], Ethikkommission der Universität Ulm, (Oberer Eselsberg) [Germany], Landesamt für Gesundheit und Soziales Berlin Geschäftsstelle der Ethik-Kommission des Landes Berlin [Germany], Ethikkommission des FB Medizin der Philipps-Universität Marburg [Germany], Ethikkommission an der Medizinischen Fakultät der Eberhard-Karls-Universität und am Universitätsklinikum Tübingen [Germany], Ethikkommission an der Med. Fakultät der HHU Düsseldorf [Germany], The University of Kansas Medical Center Human Research Protection Program [US], Oregon Health & Science University Independent Review Board [US], Northwestern University Institutional Review Board [US], Spectrum Health Human Research Protection Program [US], The University of Vermont Committees on Human Subjects [US], Beth Israel Deaconess Medical Center Committee on Clinical Investigations, New Procedures and New Forms of Therapy [US], Vanderbilt Human Research Protection Program Health [US], University of Maryland, Baltimore Institutional Review Board [US], University of Southern California Institutional Review Board [US], Columbia University Medical Center Institutional Review Board [US], University of Southern California San Francisco Institutional Review Board [US], University of Pennsylvania Institutional Review Board [US] and HCA - HealthOne Institutional Review Board [US]).<br><br>PPMI: This study was conducted in accordance with the Declaration of Helsinki and the Good Clinical Practice (GCP) guidelines after approval of the local ethics committees of the participating sites.

Note that full information on the approval of the study protocol must also be provided in the manuscript.

Field-specific reporting

Please select the one below that is the best fit for your research. If you are not sure, read the appropriate sections before making your selection.

☒ Life sciences ☐ Behavioural & social sciences ☐ Ecological, evolutionary & environmental sciences

For a reference copy of the document with all sections, see nature.com/documents/nr-reporting-summary-flat.pdf

Life sciences study design

All studies must disclose on these points even when the disclosure is negative.

Sample size	This was an exploratory study that retrospectively used data from PASADENA and PPMI.<br>PASADENA: a sample of 100 participants per trial group (placebo, prasinezumab 1500 mg, prasinezumab 4500 mg) was calculated to detect a 3-point difference in the change from baseline to week 52 in the sum of scores on Parts I, II, and III of the MDS-UPDRS (primary

	end point) for the pairwise comparison of each active-treatment group with the placebo group, under the assumption of an increase of 8 points per year in the placebo group at a two-sided alpha significance level of 20% (80% power).<br>PPMI: A disease-modeling quantitative approach (weighting with propensity scores) was used to match the PPMI cohort with the PASADENA cohort.
Data exclusions	PASADENA: data from all patients who entered the open-label extension were included.<br>PPMI: This cohort was matched with the PASADENA cohort. Details of attrition of the data sample are provided in Supplementary Figure S4.
Replication	The PPMI results were aligned with previous publications (Holden SK, et al. Mov Disord Clin Pract 2018;5(1):47-53). The programming of all results was validated by different personnel involved in the activity working independently. All the work is reproducible.
Randomization	PASADENA was set up with randomizazion. This set of analyses is retrospective based on secondary data use for PASADENA. Propensity score method was applied in order to mimic randomization with the PPMI data.
Blinding	The double-blind period of PASADENA, referred to as Part 1, was already disclosed in 2022 (Pagano G, et al. New Eng J Med 2022; 387:421-431)

Reporting for specific materials, systems and methods

We require information from authors about some types of materials, experimental systems and methods used in many studies. Here, indicate whether each material, system or method listed is relevant to your study. If you are not sure if a list item applies to your research, read the appropriate section before selecting a response.

Materials & experimental systems

n/a	Involved in the study
☐	☒ Antibodies
☒	☐ Eukaryotic cell lines
☒	☐ Palaeontology and archaeology
☒	☐ Animals and other organisms
☐	☒ Clinical data
☒	☐ Dual use research of concern
☒	☐ Plants

Methods

n/a	Involved in the study
☒	☐ ChIP-seq
☒	☐ Flow cytometry
☒	☐ MRI-based neuroimaging

Antibodies

Antibodies used	Prasinezumab is an immunoglobulin class G1 (IgG1) humanized monoclonal antibody (mAb) directed against an epitope in the C-terminus of human alpha-synuclein.
Validation	N/A

Clinical data

Policy information about clinical studies
All manuscripts should comply with the ICMJE guidelines for publication of clinical research and a completed CONSORT checklist must be included with all submissions.

Clinical trial registration	PASADENA Phase II study, ClinicalTrials.gov identifier: NCT03100149
Study protocol	https://www.nejm.org/doi/suppl/10.1056/NEJMoa2202867/suppl_file/nejmoa2202867_protocol.pdf
Data collection	PASADENA: Patients were enrolled from June 2017 to November 2018 in centers located in USA, France, Austria, Germany, Spain.<br>PPMI: Patients were enrolled from July 2010 to May 2013 in centers located in USA, France, Austria, Germany, Spain, Austria, Greece, Israel, Italy, Norway, United Kingdom.
Outcomes	The primary endpoints were: the change from baseline to year 4 in the severity of motor progression (irrespective of starting of symptomatic treatment) as measured by the change in the MDS-UPDRS Part II, Part III in ON- and OFF-state, motor subscores (bradykinesia, rigidity, resting tremor), axial signs, tremor motor severity subscore, and non-tremor motor severity subscore in PASADENA 4 years OLE participants compared to the propensity score-weighted PPMI cohort.<br>The secondary and exploratory endpoints were: the change from baseline to year 4 in LEDD, MDS-UPDRS Part I sleep-related subscores (i.e., items 7 and 8 for sleep and item 13 for fatigue), the severity of motor complications as measured by MDS-UPDRS Part IV, and DaT-SPECT in the bilateral putamen and bilateral caudate (secondary), and the odds of having H&Y ≥3 versus <3 (exploratory) in PASADENA 4 years OLE participants compared to the PPMI cohort.

Plants

Seed stocks	N/A
Novel plant genotypes	N/A
Authentication	N/A
